# New Mode of Treating Intestino-Vaginal Fistula

**Published:** 1830-01-01

**Authors:** 


### Case of Artificial Anus opening into the Vagina; new Mode of Treatment. By M. Casamayor.*

On the 3d of March, 1826, M. Casamayor was requested to see a female, 42 years of age, residing in a village of the canton of Saint Mary in the Lower Pyrennees. After six natural labours, she had been brought to bed on the 4th of May, 1822, about the fifth month of her pregnancy. The pains were violent, the loss of blood considerable, and ten minutes after the expulsion of the fœtus, there issued from the vagina a fold of intestine, along with the placenta and much coagulum. The portion of gut extended down to the middle of the thighs, soon inflamed throughout its whole extent, and finally sloughed at the most depending part, whence extremely fetid stercoraceous matters escaped. A village surgeon was sent for, but not being able or willing to come, the patient, with the object of compelling the fæcal matters to resume their proper channel, tied a ligature round the protruding intestine, as high as she could get at it. On the sixth day the strangulated gut sloughed off, and for twenty days afterwards fæcal, bilious, and mucous matters, occasionally blood, and pus in great quantity were passed by the vagina. The patient at first was extremely reduced, but at length she gradually plucked up her embonpoint, although the fæces were always voided per vaginam, the operation generally occupying about a quarter of an hour, and occurring two hours and a half after a

* Journ. Hebdomad. No. 43.

meal. Sometimes once a month, sometimes once in two months, some excrement, resembling well-chewed liquorice root smeared with white of egg, made its exit from the anus.

Such was the account which M. Casamayor received, and he next proceeded to institute a local examination. The mucous membrane of the vulva and inferior half of the vagina was yellowish, wrinkled, thickened and almost cartilaginous in several points. About an inch from the neck of the uterus, the vagina presented a considerable stricture, immediately beneath which was a circular opening, sufficiently large to admit the end of the forefinger, and giving issue to soft fæces, in the posterior part and left side of the passage. Having introduced the left fore-finger into this opening, and the right into the rectum, they were found to be separated from each other by a fleshy, moveable mass, of the thickness and consistence of an ordinary umbilical cord. This cord, *cordon,* descended from the left iliac region in a serpentine manner and terminated at the inferior edge of the intestino-vaginal opening. In order to ascertain yet more clearly, whether any communication existed between the rectum and the upper part of the intestine, an enema was administered, but it all returned in the course of ten minutes by the anus, not a drop escaping through the vaginal aperture.

From these particulars M. Casamayor reasonably enough concluded, that the intestine which had protruded through the laceration in the vagina was the ileum—that the protruding portion having sloughed off under the ligature, the two extremities in contact with the vaginal opening had united in that situation, in consequence of inflammation—and lastly, that the upper extremity of the gut had remained pervious to the issue of fæces, whilst the lower had degenerated into a consolidated cord.

After much hesitation, and a delay of upwards of a year from his first visit, our author was prevailed on to attempt some operation, in order to release the patient from her dreadful malady. His object was to establish a communication between the ileum and rectum, above the artificial opening in the anus, so that the fæces might find a ready passage by the former.

He provided himself with steel forceps having separate branches. The forceps was about ten inches and a half in length; the branches, which are distinguished by the terms male and female, were cylindrical, the diameter of a thick writing quill, and curved for about three quarters of their extent. The surfaces which looked towards each other were concave, and comprised between them an oblong space nearly eight inches long, and an inch and a quarter broad, where the utmost separation was effected. They terminated insensibly; on the one hand—in a plane, oval (bite) *mors* grooved crossways, eight lines in its long diameter, corresponding to that of the instrument, and four in its short or transverse diameter; on the other hand—in a straight prolongation, two inches and a half in length, flattened from within outwards, three lines in thickness and five in breadth.* The prolongation of the female branch presented in the direction of its length three holes, situated eight lines from each other and adapted to receive three nails fixed in the corresponding parts of the prolongation of the male branch. At the distance of an inch from the prolongation *(à un pouce du prolongement)* the two branches became thicker, and in the centre of this enlarged part the female branch had a hole, and the male a worm, through which passed a screw, for the purpose of approximating the two and graduating the pressure. The *mors* of the forceps were covered with a fine skin as well as the part of the branches comprised between them and the screw. Such is the description of the instrument, translated as well as our comprehension of the French technical terms would permit. Though we candidly confess our inability to understand

* It seems evident to us that the *mors* answers to the ordinary rough part of the common forceps, and the *prolongement* to the handle.—*Rev.*

some of the minor points, yet we see sufficient to enable us to recognize a great similarity between the present instrument and that invented by M. Dupuytren for the treatment of artificial anus. But this by the way.

On the 21st of April the patient was restricted to barley water and lemonade and several injections were thrown up the vagina. On the next day, the instrument was applied. The patient being placed in a convenient position on the bed with the thighs held apart, the forceps were smeared with cerate, and the female branch held by the "prolongation" in the right hand was conducted on the left index finger into the ileum for the space of an inch, through the artificial opening. The branch thus situated was confided to an assistant. The male branch held also by the "prolongation" but with the left hand, was then introduced along the right index finger into the rectum, to the same height as the female branch occupied in the ileum. The branches were then joined together, and given to the assistant to keep firmly in their place. M. Casamayor now introduced the fore-fingers into the rectum and ileum, and discovered that the *mors* of the forceps in either gut were placed about an inch above the artificial opening, that they exactly corresponded, and that they embraced only the walls of the ileum and rectum, the fleshy cord which intervened between them being pushed aside towards the left. Satisfied of these circumstances, the branches of the forceps were closed till pain was experienced by the patient, and secured by a T bandage.

Symptoms of inflammation of the bowels and inferior part of the peritoneum appeared in the night, but not to so high a degree as to cause any alarm. On the next day the branches were closed to the utmost possible extent, the patient bled, and the belly fomented. On the 25th the pain had almost subsided, and the fever disappeared, whilst a flow of mucous, bilious, and purulent matters had taken place along the branches of the forceps both in the rectum and vagina. On the 26th, the patient was comfortable, and the instrument was taken out. The portion of the intestinal parietes included between the *mors* of the branches came away with the female, and thus established a direct communication between the cavity of the ileum and rectum. By the first of May the suppuration had entirely ceased, and fæcal matters passed in greater quantity by the anus than by the vagina. On examination, the opening between the ileum and rectum was found to be situated about an inch from the vaginal aperture, nearly circular, sufficiently large to allow two fingers to pass readily from one intestine to the other, and with edges apparently cicatrized.

On the 8th, the patient having recovered her strength, our enterprising surgeon endeavoured to heal the vaginal aperture, by introducing a hollow bougie into the rectum, in order that the fæces might find a free passage by the tube. The next day, however, he was compelled to withdraw it, in consequence of the inconvenience it produced. On the 10th another expedient was tried, but with the same ill effects as the former, and it also was abandoned. On the 12th a third plan was had recourse to with no better effect than the preceding, and on the 13th the fourth and last was attempted. The pain produced in the hypogastrium was so severe, that M. Casamayor determined to abandon the remainder of the cure to Nature, in the hope that she would accomplish the final obliteration of the vaginal fistula without any further assistance from art.

The patient was very well content with what had been already done, and prepared to quit St. Mary for her native village. Having exposed herself, however, naked and perspiring, to a current of cold air, she was suddenly attacked with a violent pleuro-pneumony, under which she sank in the course of four days. No examination of the body was permitted by the parents.

The above is really a very interesting case, and although the final result was so unfortunate, the details redound to the ingenuity and perseverance of the surgeon. It is not at all improbable that had the patient lived, the vaginal fistula would have closed, or become greatly reduced in size. As it was, the re-establishment of the anal passage, even so far as it went, was a point of no mean importance to be accomplished in so short a space of time. The method of proceeding, was exactly similar in principle, and indeed in application, to that of M. Dupuytren in ordinary cases of artificial anus, and the inconveniences so slight, that English surgeons may derive an useful hint from its perusal.

